# Supplementary material for: An Arg/Ala-rich helix in the N-terminal region of M. tuberculosis FtsQ is a potential membrane anchor of the Z-ring
Source: Commun Biol. 2023 Mar 23;6:311. doi: 10.1038/s42003-023-04686-5 (PMC10036325; doi:10.1038/s42003-023-04686-5)
Supplement: Supplementary file 2 — Supplementary Tables and Figures [file 42003_2023_4686_MOESM2_ESM.pdf]

An Arg/Ala-Rich Helix in the N-Terminal Region of *M. tuberculosis* FtsQ Is a Potential Membrane Anchor of the Z-ring

Sean T. Smrt[1,2,6], Cristian A. Escobar[1,3,6], Souvik Dey[4], Timothy A. Cross[1,2,3,*], and Huan-Xiang Zhou[4,5,*]

[1]National High Magnetic Field Laboratory, Tallahassee, FL 32310, USA

[2]Department of Chemistry and Biochemistry, Florida State University, Tallahassee, FL 32306, USA

[3]Institute of Molecular Biophysics, Florida State University, Tallahassee, FL 32306, USA

[4]Department of Chemistry, University of Illinois Chicago, Chicago, IL 60607, USA

[5]Department of Physics, University of Illinois Chicago, Chicago, IL 60607, USA

[6]These authors contributed equally.

*Correspondence should be addressed to H.X.Z. (e-mail: hzhou43@uic.edu) or T.A.C. (e-mail: timothyacross@gmail.com).

Supplementary Material

Supplementary Table 1. Chemical shifts assignments of FtsQ1-99

Residue number	Residue name	N	HN	CO	Cα	Cβ
1	M	119.1	8.346	176.4	55.54	32.52
2	T	114.5	8.011	174.4	61.75	69.64
3	E	122.8	8.319			
4	H	119.9	8.352			
5	N	121.0	8.390	174.7	52.99	39.06
6	E	121.3	8.456	175.7	56.09	30.19
7	D	122.9	8.370			40.76
8	P			177.0	63.21	31.90
9	Q	119.8	8.492	175.9	55.72	28.86
10	I	121.2	7.916	175.9	61.02	38.57
11	E	124.8	8.367	176.0	56.37	30.10
12	R	123.0	8.285	176.0	55.68	30.78
13	V	122.1	8.209	175.7	61.99	32.54
14	A	127.8	8.368	177.3	52.30	19.21
15	D	120.1	8.266	176.0	54.24	41.04
16	D	120.5	8.223	176.0	54.12	40.93
17	A	123.9	8.105	177.5	52.48	18.97
18	A	122.8	8.136	177.5	52.47	19.02
19	D	119.5	8.188	176.4	54.29	41.03
20	E	121.2	8.277	176.5	56.71	30.20
21	E	121.2	8.322	176.1	56.42	30.04
22	A	124.8	8.151	177.5	52.32	18.98
23	V	119.2	8.056	176.2	62.11	32.51
24	T	117.8	8.140	174.1	61.49	69.74
25	E	124.6	8.279	174.2	54.20	29.68
26	P			176.7	62.97	31.80
27	L	122.0	8.303	177.3	54.93	42.22
28	A	125.1	8.306	177.8	52.45	18.92
29	T	112.6	7.991	174.5	61.74	69.56
30	E	123.0	8.332	176.2	56.43	30.16
31	S	117.1	8.325	174.4	58.08	63.60
32	K	123.4	8.371	176.0	56.15	32.78
33	D	120.9	8.258	175.6	54.04	41.16
34	E	122.1	8.136	174.3	54.11	29.75
35	P			176.6	63.08	31.80
36	A	123.9	8.330	177.5	52.27	19.16
37	E	119.3	8.233	175.8	56.38	30.26
38	H	121.7	8.349	176.1	56.41	30.09
39	P			177.1	63.67	31.86
40	E	120.4	9.091	176.4	56.98	29.43
41	F	119.8	8.006	175.4	57.12	39.40
42	E	122.2	8.184	176.3	56.49	30.53
43	G	110.1	8.156	172.4	45.20	
44	P			178.2		
45	R	120.3	8.315			
46	R	119.8	8.292			
47	R	121.4	8.249			
48	A	123.3	8.153	179.0	53.61	
49	R	119.5	8.091	177.6		
50	R	121.9	8.232	177.3	57.58	30.08
51	E	120.8	8.466	178.0	58.02	29.71
52	R	120.5	8.193	177.5	57.94	30.23
53	A	123.7	8.126	179.6	53.96	18.38
54	E	119.9	8.445	178.3	57.93	29.50
55	R	121.4	8.187	178.0	57.93	29.66
56	R	120.7	8.195	178.0	57.81	29.95
57	A	123.6	8.172	179.2	53.78	18.28
58	A	122.0	8.138	179.7	53.93	18.34
59	Q	119.2	8.165	177.3	57.21	28.60
60	A	123.8	8.144	179.4	53.86	18.28
61	R	119.6	8.080	177.3	57.60	30.22
62	A	122.9	8.067	179.7	53.96	18.37

63	T	114.4	8.241	175.4	64.23	69.23
64	A	125.0	8.088	180.3	54.47	18.15
65	I	119.9	8.037	177.9	63.62	38.00
66	E	122.1	7.984	178.7	58.47	29.13
67	Q	119.2	8.413	178.2	58.14	27.55
68	A	122.9	8.005	179.9	54.20	18.02
69	R	119.8	8.122	178.4	58.28	30.10
70	R	119.9	8.080	177.9	58.14	30.16
71	A	122.7	8.040	178.8	53.69	18.35
72	A	121.4	7.921	179.0	53.55	18.42
73	K	119.2	7.905	177.4	57.21	32.46
74	R	120.4	7.966	177.0	57.03	30.42
75	R	121.1	8.109	176.5	56.68	30.55
76	A	124.3	8.092	177.9	52.56	18.89
77	R	119.6	8.154	176.9	56.44	30.46
78	G	109.1	8.284	173.9	45.13	
79	Q	119.8	8.104	175.7	55.58	29.41
80	I	122.5	8.196	176.3	61.01	38.35
81	V	124.8	8.250	175.9	62.10	32.57
82	S	119.6	8.317	174.4	58.09	63.74
83	E	123.1	8.425	176.2	56.58	30.12
84	Q	121.0	8.323	175.3	55.67	29.29
85	N	120.5	8.446	173.0		
86	P			176.5	63.11	31.87
87	A	123.4	8.231	177.4	52.13	18.91
88	K	121.4	8.086	174.2	53.86	32.38
89	P			176.6	62.95	31.86
90	A	124.4	8.348	177.4	52.12	19.02
91	A	123.6	8.231	177.3	52.21	19.13
92	R	120.3	8.270	176.7	56.05	30.64
93	G	110.0	8.332	173.7	45.07	
94	V	119.7	7.948	176.1	62.17	32.54
95	V	125.1	8.240	175.9	62.16	32.48
96	R	125.8	8.433	176.4	55.96	30.75
97	G	110.2	8.369	173.7	45.04	
98	L	122.1	8.078	176.4	55.19	42.37
99	K	126.4	7.808	181.0	57.47	33.62

Supplementary Table 2. Differences in average chemical shifts between helical and nonhelical positions

	Helical			Nonhelical		
**Residue type**	**C^O**	**C_α**	**C_β**	**C^O**	**C_α**	**C_β**
Ala (7[a] vs. 9[b])	179.7 ± 0.3[c]	54.0 ± 0.2	18.3 ± 0.1	177.5 ± 0.1	52.3 ± 0.1	19.0 ± 0.1
Arg (7[a] vs. 5[b])	177.8 ± 0.4	57.9 ± 0.2	30.1 ± 0.2	176.5 ± 0.3	56.2 ± 0.4	30.6 ± 0.1
Glu (3[a] vs. 11[b])	178.3 ± 0.3	58.1 ± 0.2	29.4 ± 0.2	175.8 ± 0.8	56.1 ± 0.9	30.0 ± 0.3

[a]Number of this amino acid within the helical core, or residues 50-70.
[b]Number of this amino acid within nonhelical regions, or residues 1-42 and 80-99.
[c]Errors are standard deviations.

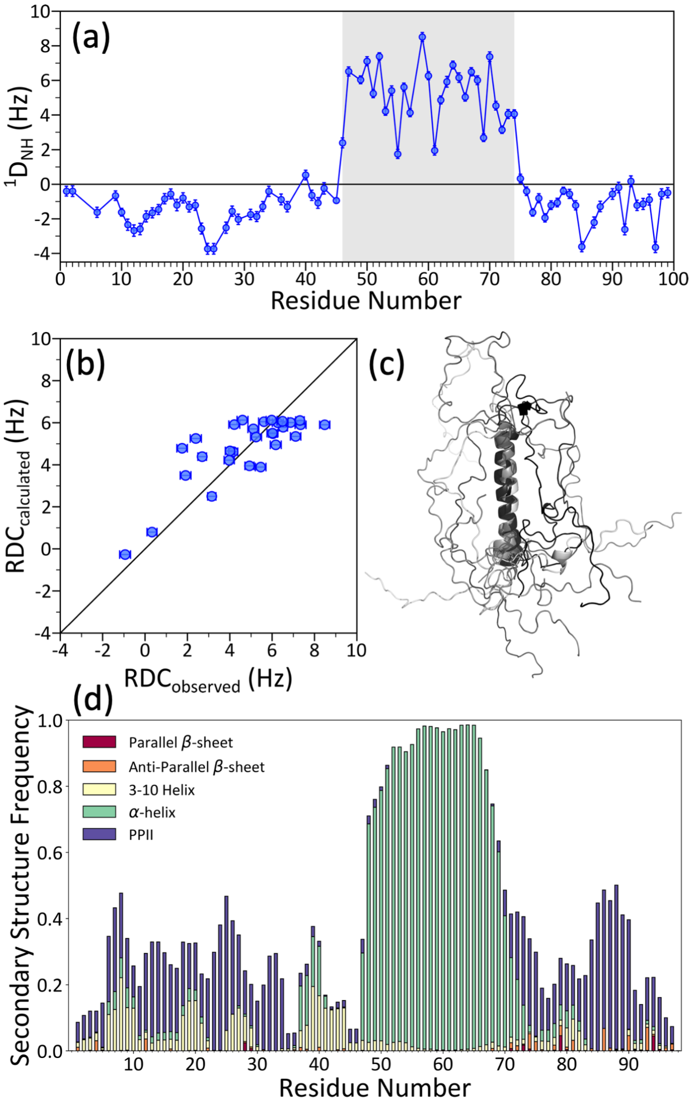

Supplementary Figure 1. Helix formation in FtsQ1-99. (a) RDCs of uniformly ^{15}N-labeled FtsQ1-99 aligned with neutral stretched acrylamide gels in 20 mM Tris (pH 6.85) with 50 mM NaCl measured at 800 MHz and 305 K. Helical residues (46-74) are highlighted in grey. Error bars represent the uncertainties in the peak positions in the spectra of isotropic and partially aligned samples. The uncertainties in peak position were estimated based on line width and peak height, and were propagated to obtain the error of DRC for each ^{15}N site. (b) Correlation of observed RDC values for residues 45-75 with those back-calculated from the lowest-energy model. (c) Eight lowest-energy models. (d) Secondary structure propensities in MD simulations.

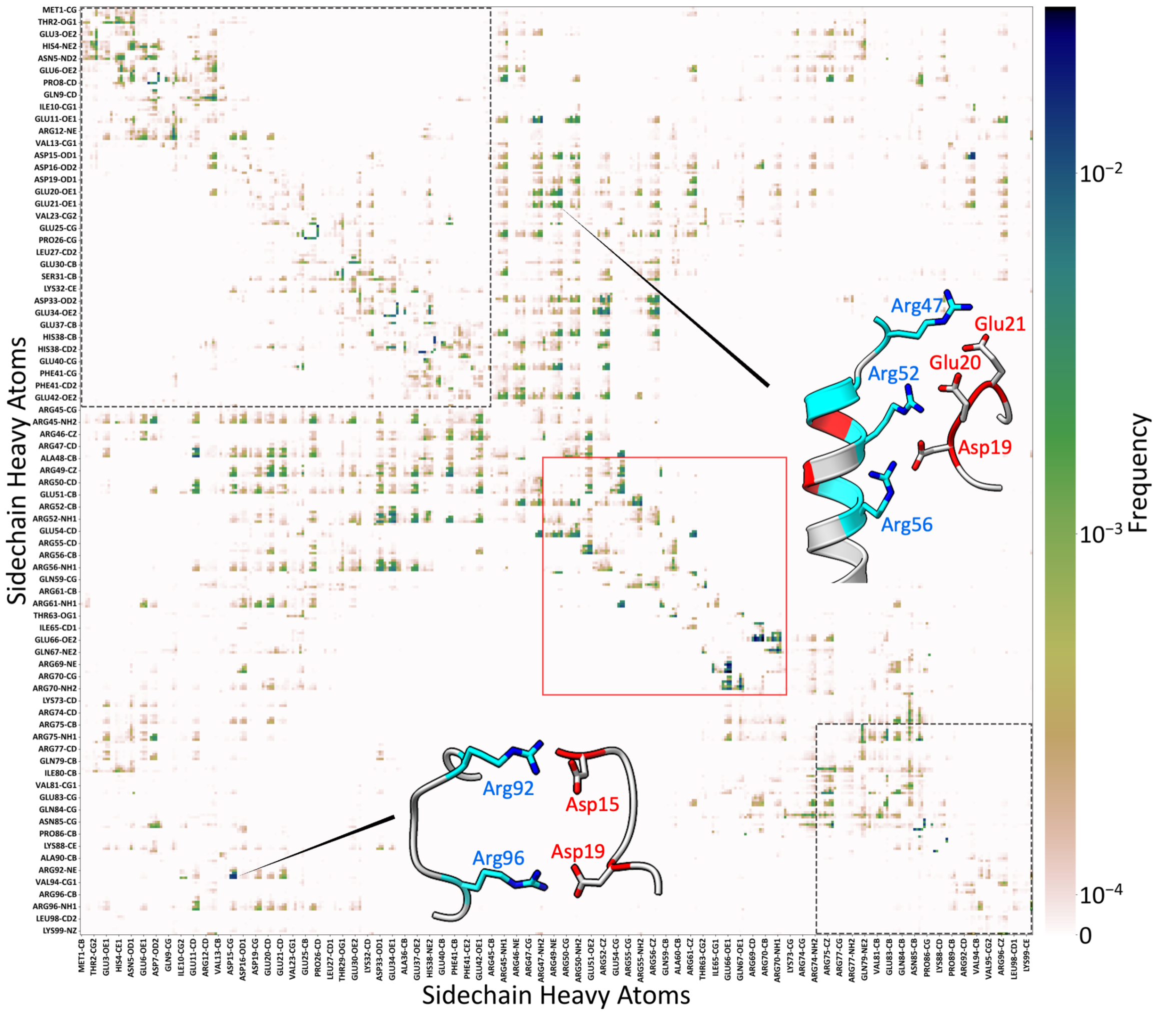

Supplementary Figure 2. Sidechain-sidechain contact map of FtsQ1-99 from MD simulations in solution. The Arg/Ala-rich helix core (residues 48-72) is indicated by a red box; the N-tail (residues 1-44) and C-tail (residues 75-99) are indicated by a black dotted box. Inset: two snapshots illustrating the contact formation of the N-tail with either the Arg/Ala-rich helix or the C-tail.

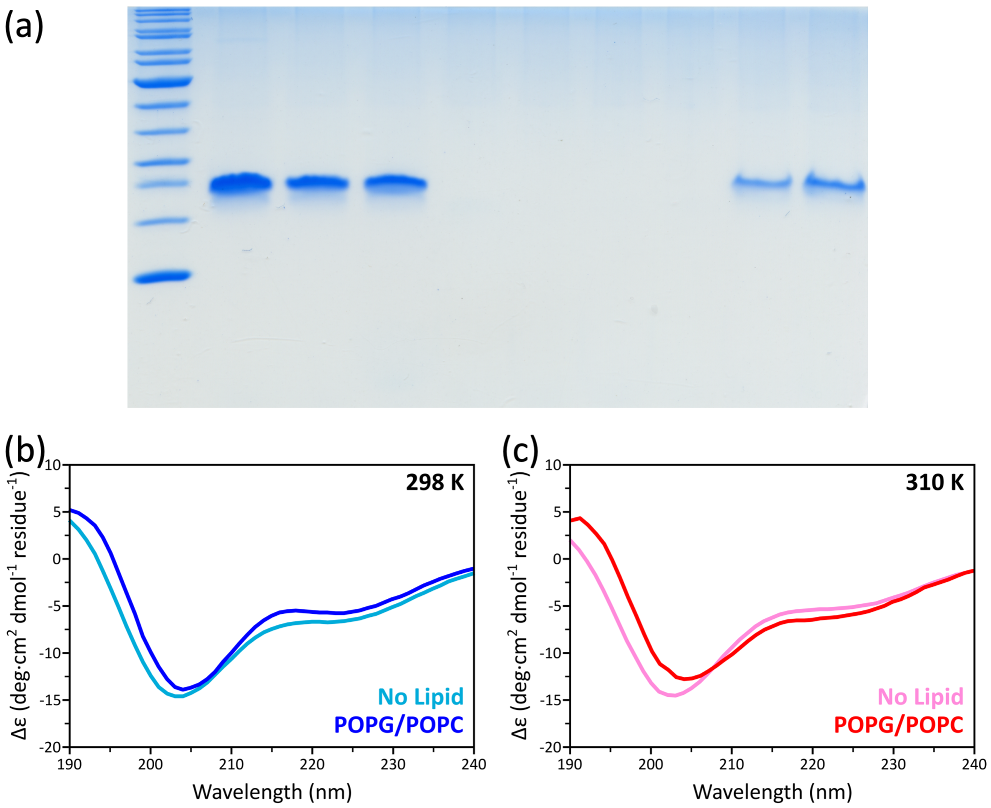

Supplementary Figure 3. SDS-PAGE gel and circular dichroism (CD) spectra of FtsQ1-99. (a) Uncropped version of the gel shown in Fig. 4a. (b) CD spectra of 20 μM FtsQ1-99 in 2 mM Tris buffer (pH 6.85) with 5 mM NaCl at 298 K. (c) Corresponding data at 310 K. The spectra were taken either in the absence (marine and pink) or presence (blue and red) of POPG/POPC lipid vesicles (7:3 molar ratio). The protein-to-lipid was 1:16.5.

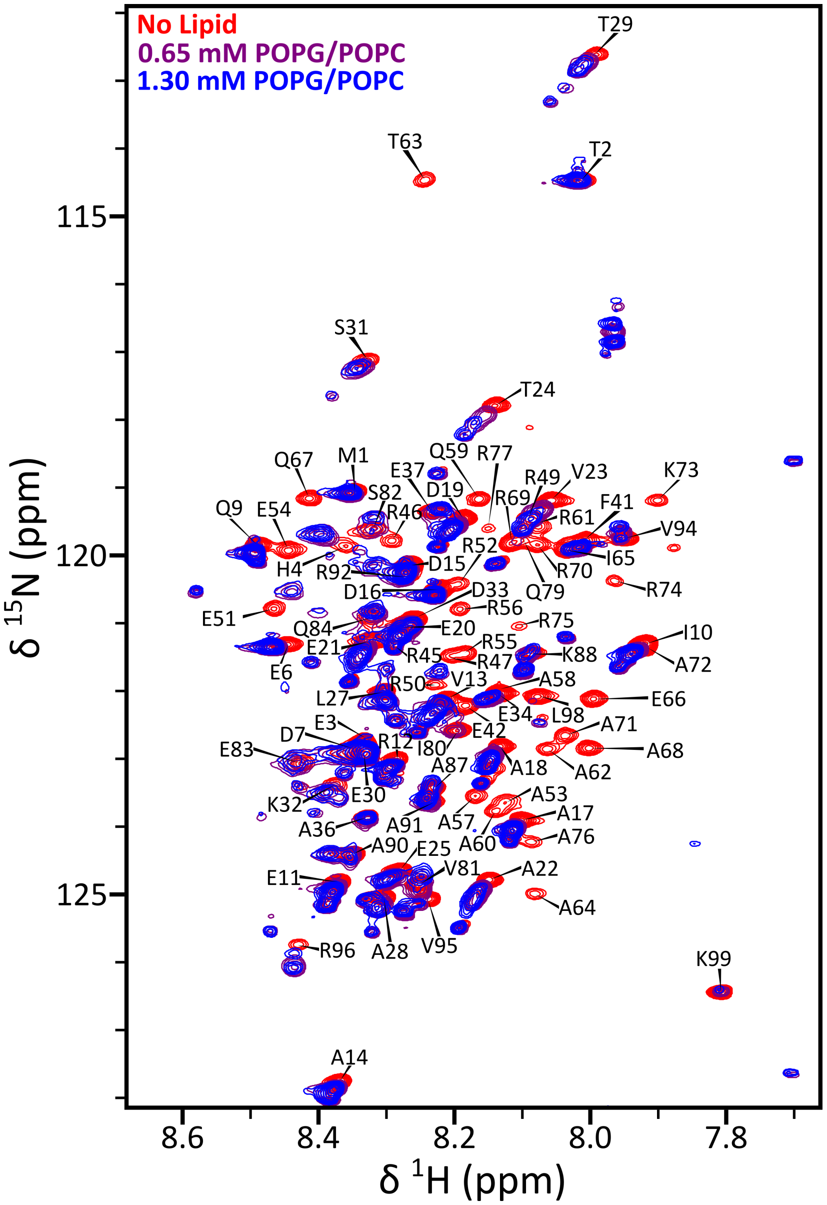

Supplementary Figure 4. ${}^{1}H$-${}^{15}N$ HSQC spectra of 130 μM uniformly ${}^{13}C$-${}^{15}N$-labeled FtsQ1-99 in the absence (red) or presence of POPG/POPC (7:3) vesicles at 1:5 (purple) or 1:10 (blue) protein-to-lipid ratios. Acquired at 800 MHz and 305 K in 20 mM Tris buffer (pH 6.85) with 50 mM NaCl.

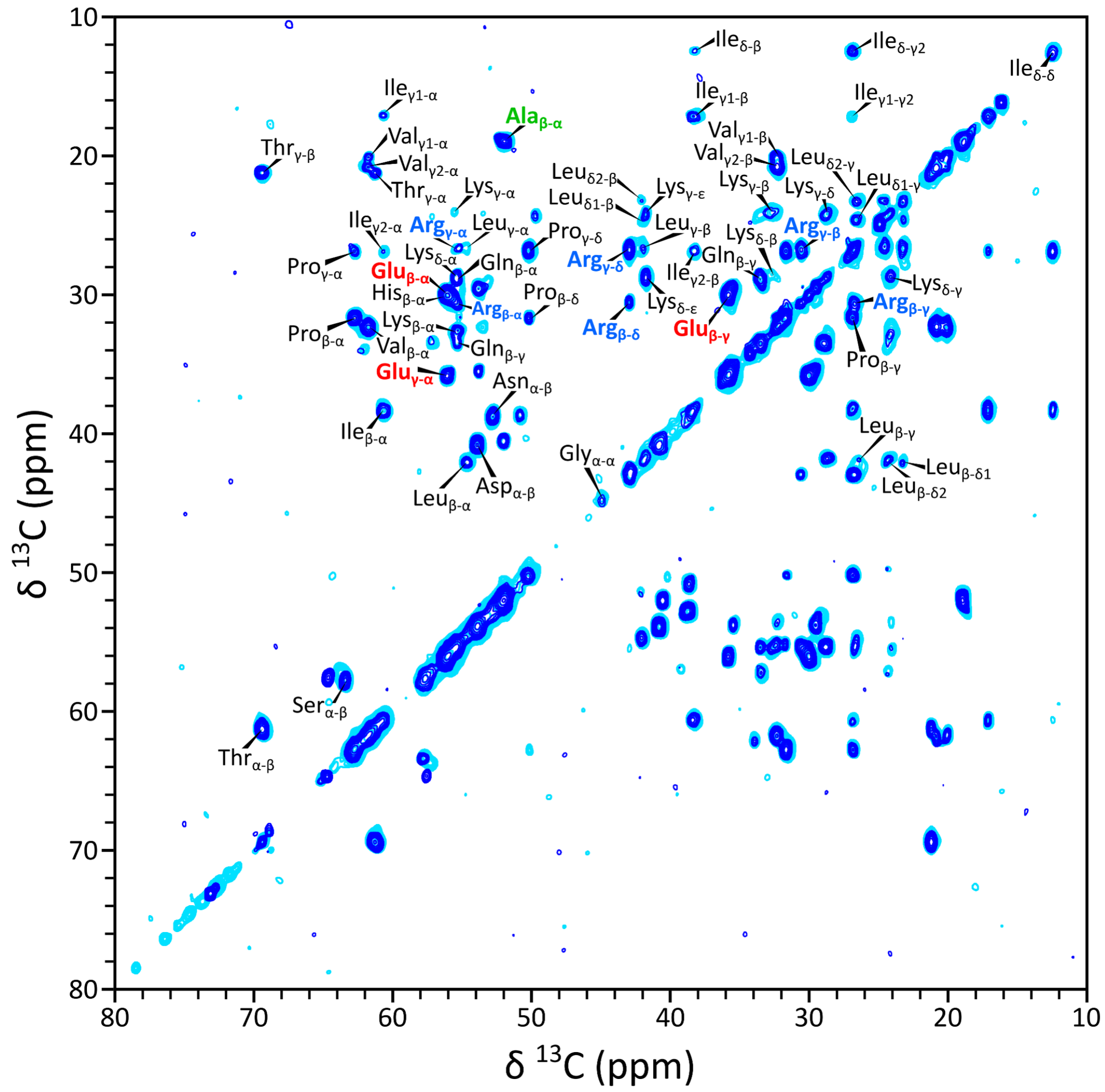

Supplementary Figure 5. TOBSY-INEPT ^{13}C-^{13}C correlation spectra of FtsQ1-99 bound to 7:3 POPG/POPC (blue) or native (cyan) lipid vesicles. Acquired at 800 MHz with a 13 kHz spinning rate at 298 K in 20 mM Tris buffer (pH 6.85) with 50 mM NaCl.

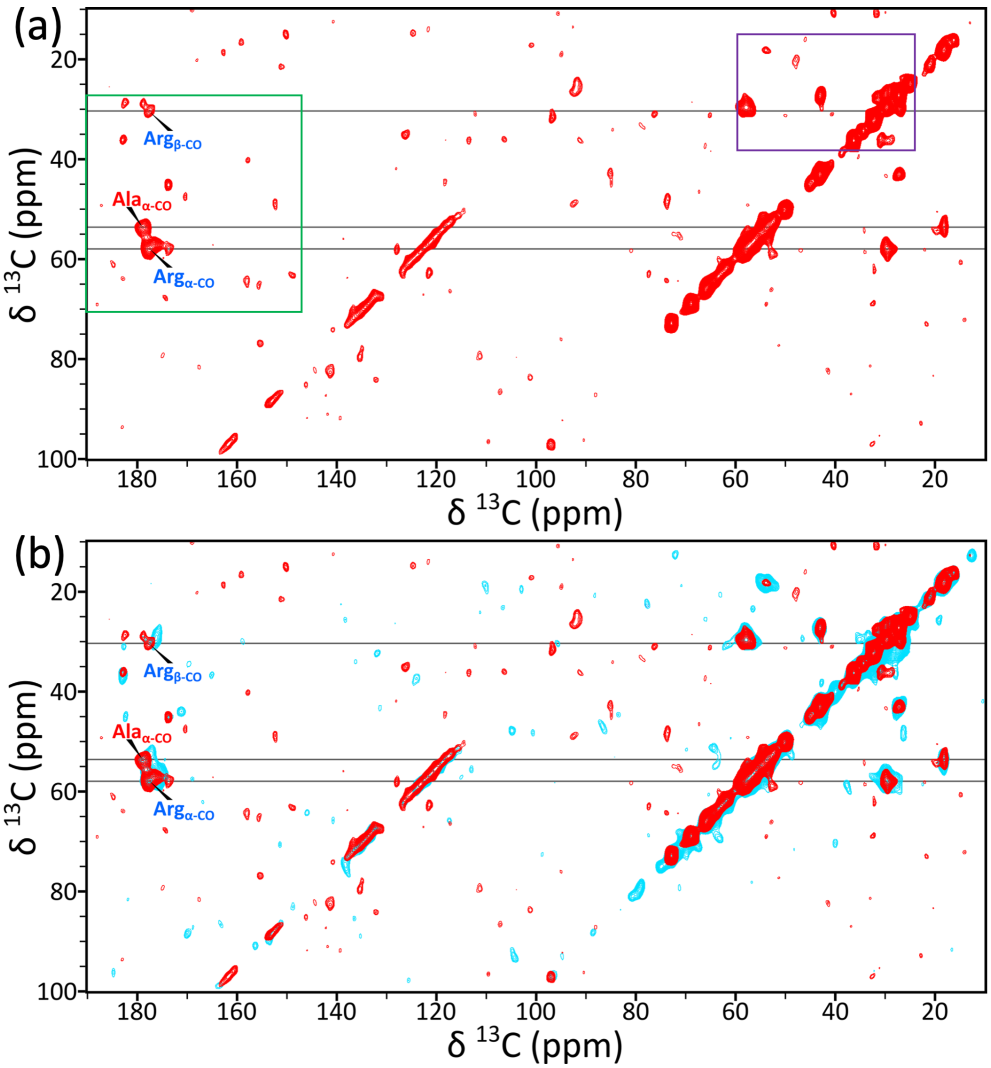

Supplementary Figure 6. CP-PARIS ^{13}C-^{13}C correlation spectra of FtsQ1-99 bound to lipid vesicles. (a) The spectrum of FtsQ1-99 bound to 7:3 POPG/POPC vesicles. The regions enclosed in the purple and green boxes are shown in Fig. 5A, B. (b) Overlay of this spectrum (red) with the counterpart using native lipid vesicles (cyan). Acquired at 800 MHz with a 13 kHz spinning rate at 285 K in 20 mM Tris buffer (pH 6.85) with 50 mM NaCl.

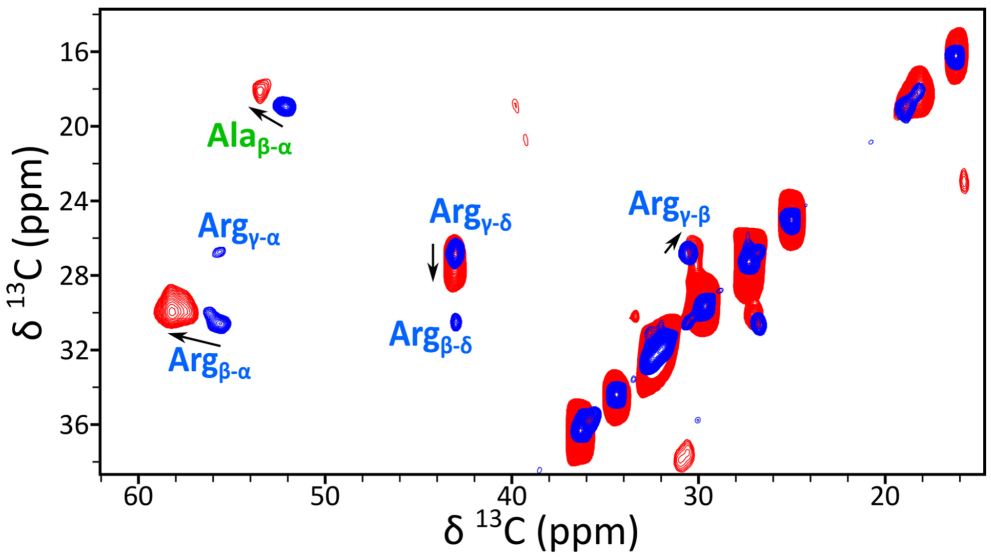

Supplementary Figure 7. Identification of comparatively rigid and dynamic residues of FtsQ1-99 by MAS solid-state NMR using Ala and Arg specifically-labeled FtsQ1-99. Overlay of INEPT (blue) and CP (red) ^{13}C-^{13}C correlation spectra. Acquired at 800 MHz with a 13 kHz spinning rate, employing 7.5 ms and 30 ms mixing times for TOBSY-INEPT (298 K) and CP-PARIS (285 K), respectively. The samples contained $^{13}C^{15}N$-[AR]-labeled FtsQ1-99 bound to 7:3 POPG/POPC vesicles at a protein-to-lipid ratio of 1:20 in 20 mM Tris buffer (pH 6.85) with 50 mM NaCl. The selected region highlights the only significant sidechain resonances, assigned to Ala and Arg residues.

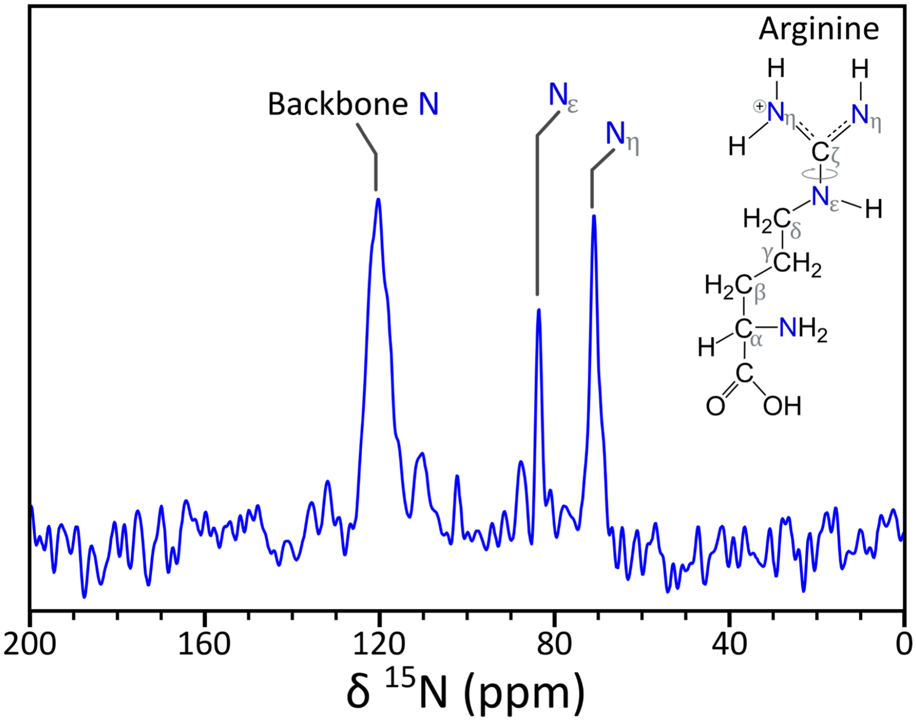

Supplementary Figure 8. One-dimensional ^{15}N CP-MAS spectrum of uniformly ^{13}C-^{15}N-labeled FtsQ1-99 bound to POPG/POPC (7:3) vesicles in 20 mM Tris buffer (pH 6.85) with 50 mM NaCl. Acquired at 600 MHz and 295 K with an 8 kHz spinning rate.

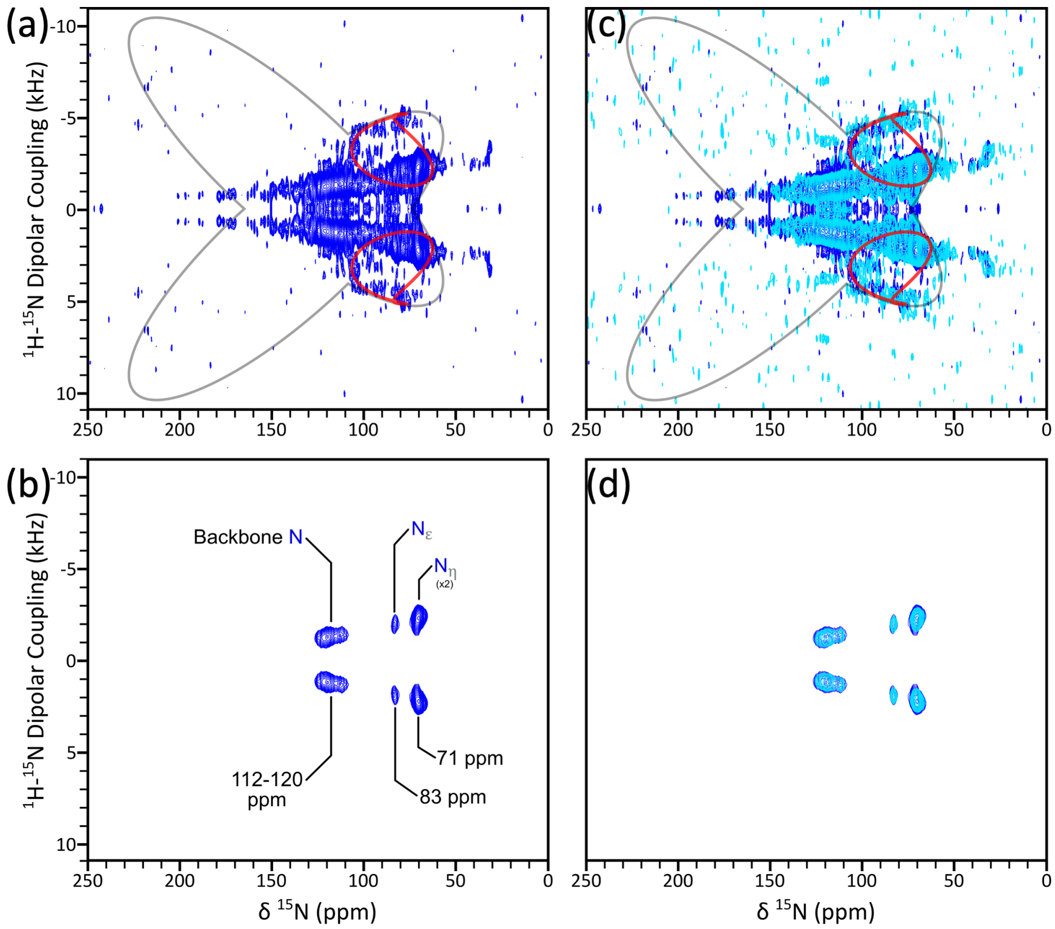

Supplementary Figure 9. "Magic-sandwich" PISEMA spectra of uniformly ^{15}N-labeled FtsQ1-99 bound to lipid bilayers mechanically oriented using glass slides. (a) Low-contour plot of the spectrum of FtsQ1-99 bound to 7:3 POPG/POPC bilayers, presenting a helical wheel with a lateral orientation relative to the bilayer surface. Also shown is a simulated powder pattern using average ^{15}N CSA tensor parameters: σ_{11} = 57.3 ppm, σ_{22} = 81.2 ppm, and σ_{33} = 228.1 (black) and a PISA helical wheel with a tilt of 74° (red). (b) High-contour plot highlighting the significant resonances assigned to Arg sidechain sites embedded in the membrane surface. The intensities spanning 112-120 ppm with modest dipolar alignment are likely from backbone nitrogens of residues flanking the helix. (c-d) Overlay with the counterparts using native lipid bilayers (cyan). Acquired at 800 MHz and 298 K on samples at a protein-to-lipid ratio of 1:40 in 5 mM Tris buffer (pH 6.85) with 2 mM NaCl.

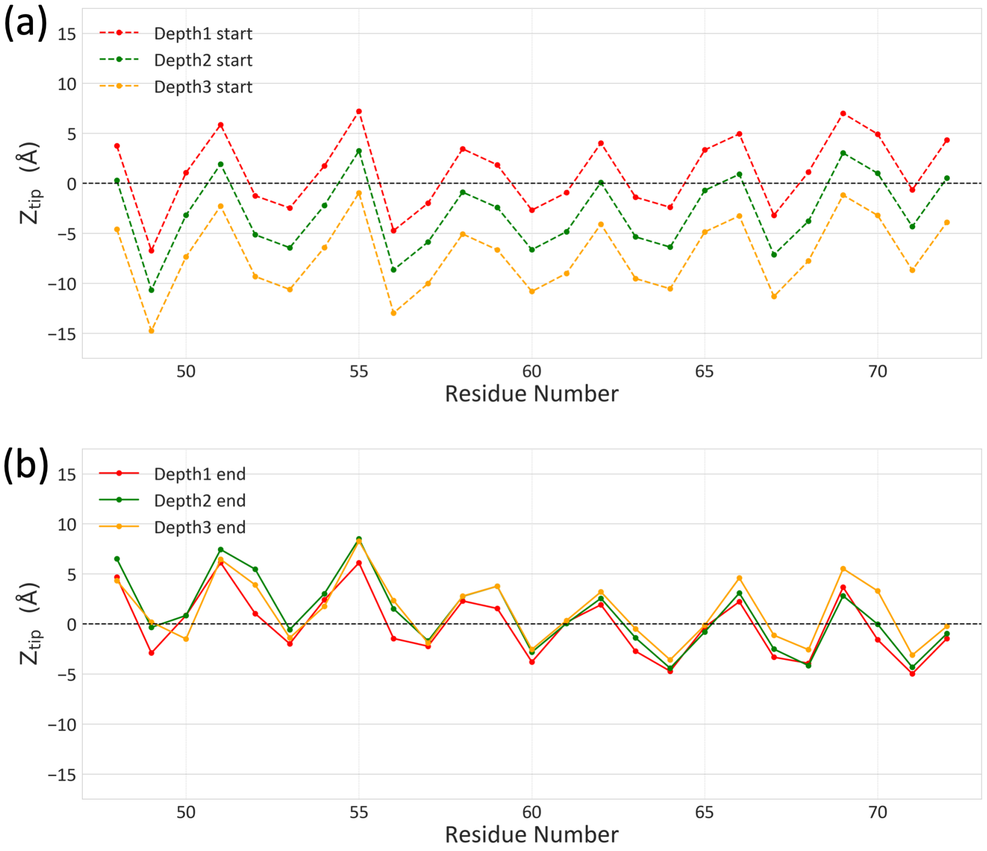

Supplementary Figure 10. Burial depth of the Arg/Ala-rich helix in membranes. (a) Z_{tip} distances of residues 48-72 sidechains in three initial structures for MD simulations. (b) Mean Z_{tip} distances in the last 10 ns of 200 ns MD simulations. The three simulations all converged to the same shallow burial depth.

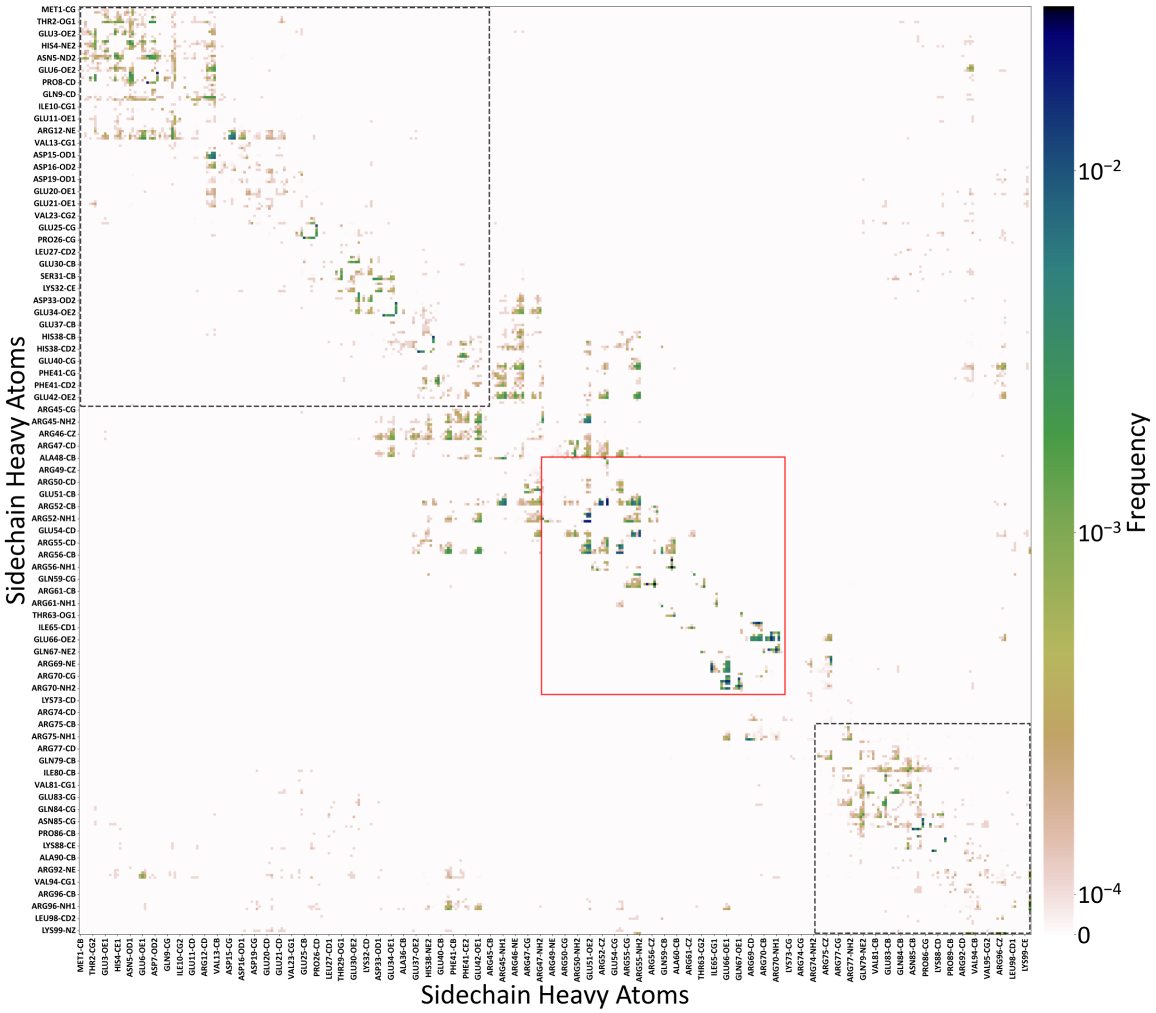

Supplementary Figure 11. Sidechain-sidechain contact map of membrane-bound FtsQ1-99 from MD simulations. The Arg/Ala-rich helix core (residues 48-72) is indicated by a red box; the N-tail (residues 1-44) and C-tail (residues 75-99) are indicated by a black dotted box.

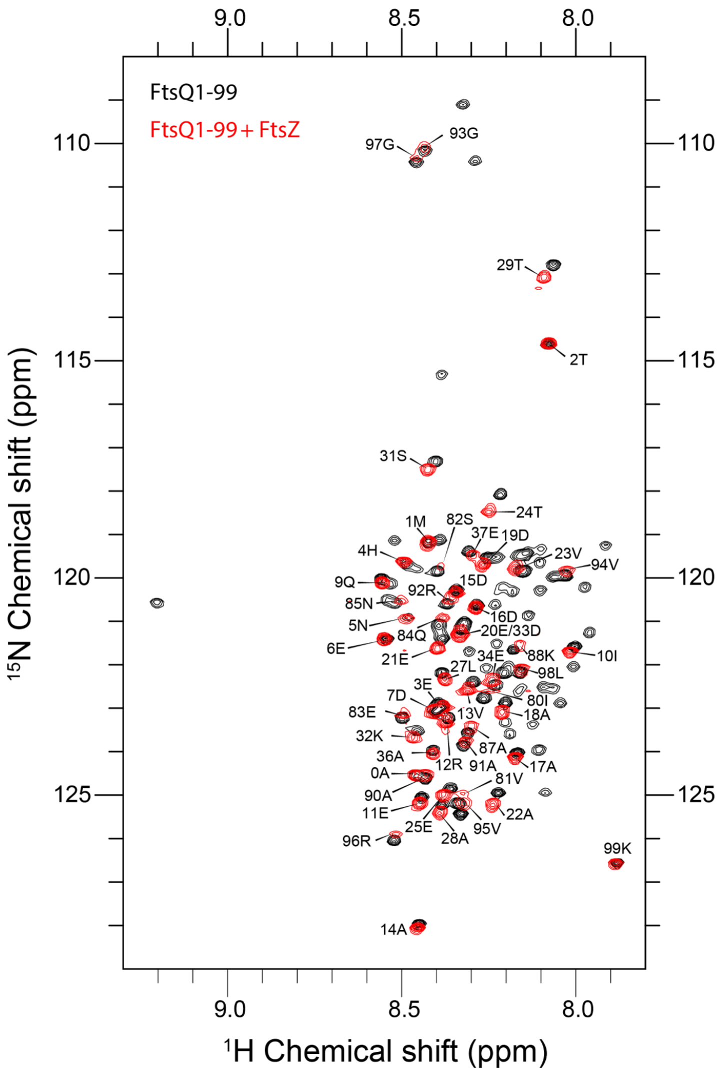

Supplementary Figure 12. ^{1}H-^{15}N HSQC spectra of 50 µM uniformly ^{13}C-^{15}N-labeled FtsQ1-99 in the absence (black) or presence (red) of 50 µM FtsZ. Acquired at 800 MHz 298 K in 20 mM phosphate buffer (pH 6.5) with 25 mM NaCl.

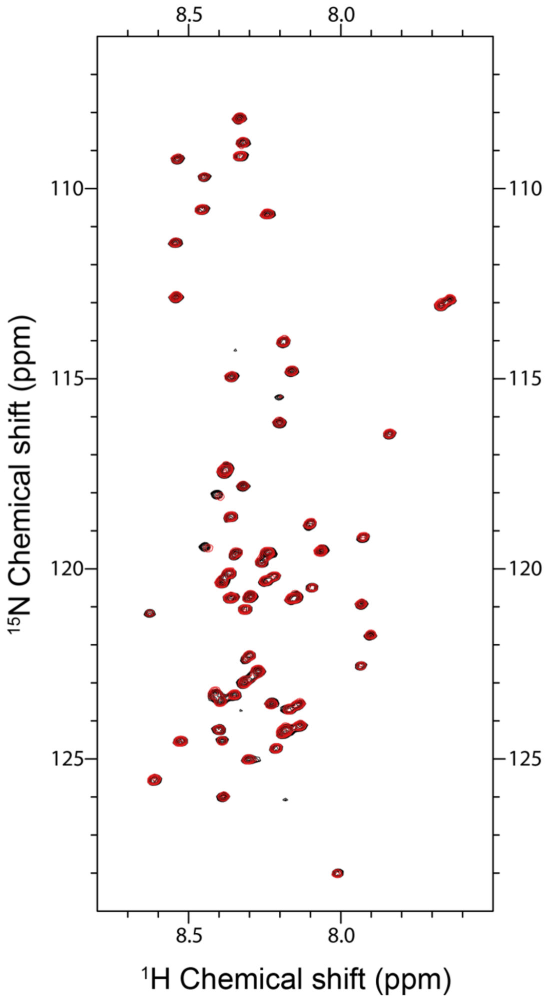

Supplementary Figure 13. ^{1}H-^{15}N HSQC spectra of 50 μM uniformly ^{13}C-^{15}N-labeled FtsZ C-tail in the absence (black) or presence (red) of 75 μM FtsQ1-99. Acquired at 800 MHz and 298 K in 20 mM phosphate buffer (pH 6.5) with 25 mM NaCl.

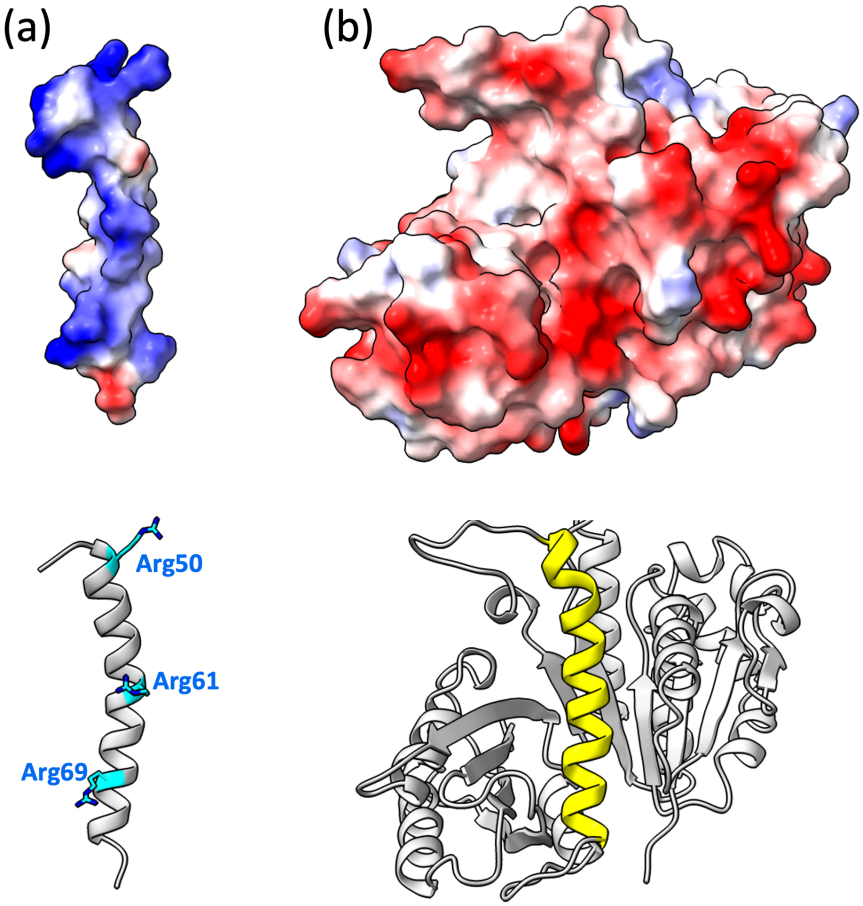

Supplementary Figure 14. Electrostatic surfaces of (a) FtsQ Arg/Ala-rich helix and (b) FtsZ GTPase domain. As illustrated by the structures in the lower panels, the view of the FtsZ GTPase domain is directly into the inter-subdomain cleft (with the H7 helix at the base of the cleft in yellow); the view of the FtsQ helix is into the face that would be buried in the modeled complex shown in Fig. 8a. On this face are Arg 50, Arg61, and Arg69, shown as sticks.
