# Supplementary material for: An Arg/Ala-rich helix in the N-terminal region of M. tuberculosis FtsQ is a potential membrane anchor of the Z-ring
Source: Commun Biol. 2023 Mar 23;6:311. doi: 10.1038/s42003-023-04686-5 (PMC10036325; doi:10.1038/s42003-023-04686-5)
Supplement: Supplementary file 3 — Description of Additional Supplementary Data [file 42003_2023_4686_MOESM3_ESM.docx]

**Description of Additional Supplementary Files**

**File name:** Supplementary Data 1

**Description:** The source data behind the graphs in the paper

**File name:** Supplementary Data 2-4

**Description:** Input, initial and final coordinate files for MD simulations in solution

**File name:** Supplementary Data 5-7

**Description:** Input, initial and final coordinate files for MD simulations at membrane surface
